# Prevalence of visual impairment and associated risk factors: a cross-sectional community-based study among children and adolescents in Ghana

**DOI:** 10.3389/fopht.2026.1829462

**Published:** 2026-05-20

**Authors:** Josephine Ampomah Boateng, Werner Eisenbarth, Sylvester Kyeremeh, Albert Kwadjo Amoah Andoh, Isaiah Osei Duah Junior, Debora Abena Baidoo, Sylvia Agyekum, Daniel Boateng, Emmanuel Antwi, Kwadwo Owusu Akuffo

**Affiliations:** 1 Department of Optometry and Visual Science, College of Science, Kwame Nkrumah University of Science and Technology, Kumasi, Ghana; 2 Department of Applied Sciences and Mechanics, Hochschule München University of Applied Sciences, Munich, Germany; 3 Department of Psychology, John R. and Kathy R. Hairston College of Health and Human Sciences, North Carolina Agricultural and Technical State University, Greensboro, NC, United States; 4 Department of Epidemiology and Biostatistics, School of Public Health, Kwame Nkrumah University of Science and Technology, Kumasi, Ghana

**Keywords:** adolescent, childhood vision loss, Ghana, pediatric eye health, sub-Saharan Africa, vision impairment

## Abstract

**Objective:**

Contrary to hospital-based estimates of visual impairment, which are influenced by care-seeking behavior and clinical referral patterns, community-based studies provide a more accurate reflection of the true population burden. There is limited evidence on childhood visual impairment in Ghana; this study therefore reports the prevalence and determinants of visual impairment in a community-based sample from the Ashanti Region of Ghana.

**Methods:**

A total of 622 participants, all aged 0–20 years, 332 of whom were females, underwent comprehensive ocular examinations, including age-appropriate visual acuity assessment, refraction, anterior segment and fundus evaluations. Visual impairment was defined as visual acuity worse than 0.3 logMAR (6/12 Snellen) in the better-seeing eye. Univariate and multivariable logistic regression analyses were used to assess factors associated with visual impairment (p ≤ 0.05).

**Results:**

Of the 622 participants recruited, 571 were included in the analysis after 51 were excluded due to incomplete visual assessment. These participants ranged from 3 months to 20 years (mean ± SD: 8.88 ± 4.11 years), comprising 53.6% females. The prevalence of presenting distance visual impairment was 6.1% (95% CI: 4.3-8.4), while near visual impairment was 10.9% (95% CI: 8.3-13.9). In the multivariable analysis, age (AOR = 0.68, 95% CI: 0.57–0.82, p < 0.001) and ethnicity (AOR = 0.10, 95% CI: 0.03–0.37, p < 0.001) were significantly associated with lower odds of distance visual impairment.

**Conclusion:**

Childhood visual impairment remains relatively high in this community-based population in Ghana. Age and ethnicity were significant determinants of distance visual impairment, highlighting important demographic influences on visual health among children and adolescents. These findings underscore the need for strengthened early detection and improved access to pediatric eye care services to reduce avoidable vision loss. Longitudinal studies are warranted to further clarify risk patterns and inform targeted intervention strategies.

## 1 Introduction

Visual impairment (VI) in children refers to reduced visual function relative to age-appropriate developmental expectations, resulting in limitations in visual performance that affect everyday functioning and participation (1). It is typically defined by measurable deficits such as reduced visual acuity, visual field constriction, impaired color vision, reduced contrast sensitivity, and/or decreased stereo acuity (2, 3). Because vision is central to early neurodevelopment, childhood VI is not merely an ocular condition but a developmental disruption with broad functional consequences (4). Even mild or uncorrected visual deficits can alter trajectories of learning and skill acquisition, underscoring the importance of early detection and intervention.

The global burden of childhood VI remains substantial and unevenly distributed, with a persistent disparity between high-income countries and low- and middle-income countries (LMICs) (5). In LMICs, this burden is exacerbated by limited access to preventive eye care, delayed diagnosis, and inadequate treatment coverage, resulting in a higher prevalence of avoidable vision loss (6). In Sub-Saharan Africa, uncorrected refractive errors and cataract remain the leading causes of childhood VI, despite being largely preventable or treatable with timely intervention (7). Globally, millions of children live with VI, the majority in resource-limited settings, where the consequences extend beyond vision to include reduced educational attainment, diminished developmental potential, and long-term productivity loss (8).

The functional impact of childhood VI is extensive and cumulative across developmental domains (9). Vision plays a foundational role in cognitive, motor, and experiential learning; therefore, impairment can delay key milestones such as language development, spatial awareness, and fine and gross motor skills (10). In school-aged children, VI is associated with reduced reading fluency, poor writing performance, limited classroom participation, and increased reliance on non-visual learning modalities, all of which contribute to suboptimal academic outcomes and elevated risk of school dropout (11). Beyond academics, affected children often experience restricted participation in play and peer interaction, leading to social isolation, reduced self-esteem, behavioral difficulties, and increased psychological distress (12). At the household level, childhood VI imposes additional caregiving demands, increasing psychosocial strain and financial burden on families (13, 14).

These challenges are amplified in low-resource settings, particularly in LMICs, where structural barriers constrain access to pediatric eye care services (15). Health systems are often characterized by shortages of trained personnel, limited diagnostic infrastructure, long waiting times, and uneven geographic distribution of services. Financial constraints further restrict access to consultation, corrective devices, and treatment (15). Consequently, many children with VI remain undiagnosed or untreated, allowing largely preventable conditions to progress into long-term disability. This perpetuates cycles of educational disadvantage and socioeconomic inequity across the life course.

In Ghana, childhood VI is an emerging public health concern alongside the rising burden of refractive errors and other non-communicable eye conditions (16, 17). However, the epidemiological evidence base remains limited, particularly with respect to population-based estimates of prevalence, causes, and associated risk factors. Most existing studies have been conducted in school-based populations (5, 18–20), thereby excluding out-of-school children who may be at higher risk of undetected VI. Other studies have been geographically restricted or focused on narrowly defined populations (21, 22), limiting generalizability. As a result, the true magnitude and distribution of childhood VI in Ghana remain insufficiently characterized, constraining evidence-based planning for equitable eye health services and early detection programs.

This study provides a community-based assessment of childhood VI in the Ashanti Region of Ghana; including both in-school and out-of-school children across diverse socioeconomic contexts, which provides a more comprehensive estimate of the burden and its associated determinants. Generating robust local evidence is essential for informing policy, strengthening early screening strategies, and improving child-focused eye health systems to reduce preventable visual disability and its long-term developmental consequences.

## 2 Materials and methods

### 2.1 Study design, population, and setting

The cross-sectional study was conducted among children and adolescents aged 0 to 20 years. These children and adolescents were selected from communities in the Ashanti Region of Ghana between March 1 and March 31, 2025. With the assistance of caregivers, children’s biographical data were collected using a structured questionnaire. In addition, visual characteristics were assessed and consolidated following standardized visual assessment procedures.

### 2.2 Study population

All children and adolescents in the Ashanti Region served as the source population.

### 2.3 Study setting

The study was conducted in the Ashanti Region, one of Ghana’s most populous and socioeconomically diverse regions, characterized by a mix of highly urbanized metropolitan areas, peri-urban growth corridors, and rural communities. The region has an estimated population of 5,440,463, of whom approximately 3,504,178 are aged 0–29 years, with a substantial proportion residing in urban and peri-urban settings. Within this context, data collection was undertaken in selected district capitals and their surrounding communities, allowing access to children from diverse environmental, socioeconomic, and educational backgrounds. This setting enabled the study to capture variability in living conditions, healthcare access, and demographic characteristics that may influence visual health outcomes.

### 2.4 Sample size determination

Due to limited prior community-based data, Cochrane’s formula was used to estimate the minimum sample size at a 95% confidence level, a 5% margin of error, and an assumed population proportion of 0.5. This yielded a minimum sample size of 384 participants. To account for potential nonresponse and incomplete data, a 5% attrition rate was added, resulting in an adjusted recruitment sample size of 404.

### 2.5 Sampling procedure

A multistage stratified sampling approach was employed to ensure representativeness across diverse ecological and socioeconomic contexts within the Ashanti Region. The 43 administrative districts were first stratified based on levels of urbanization, population density, literacy rates, and economic activity, as defined by the Ghana Statistical Service. From these strata, five districts were selected to reflect variation across highly urbanized, peri-urban, and rural settings, including Oforikrom, Afigya Kwabre South, Atwima Kwanwoma, Bosome Freho, and Juaben Municipal. Within each selected district, communities surrounding district capitals were included, and participants were recruited through community mobilization and voluntary participation.

**Figure 1:**
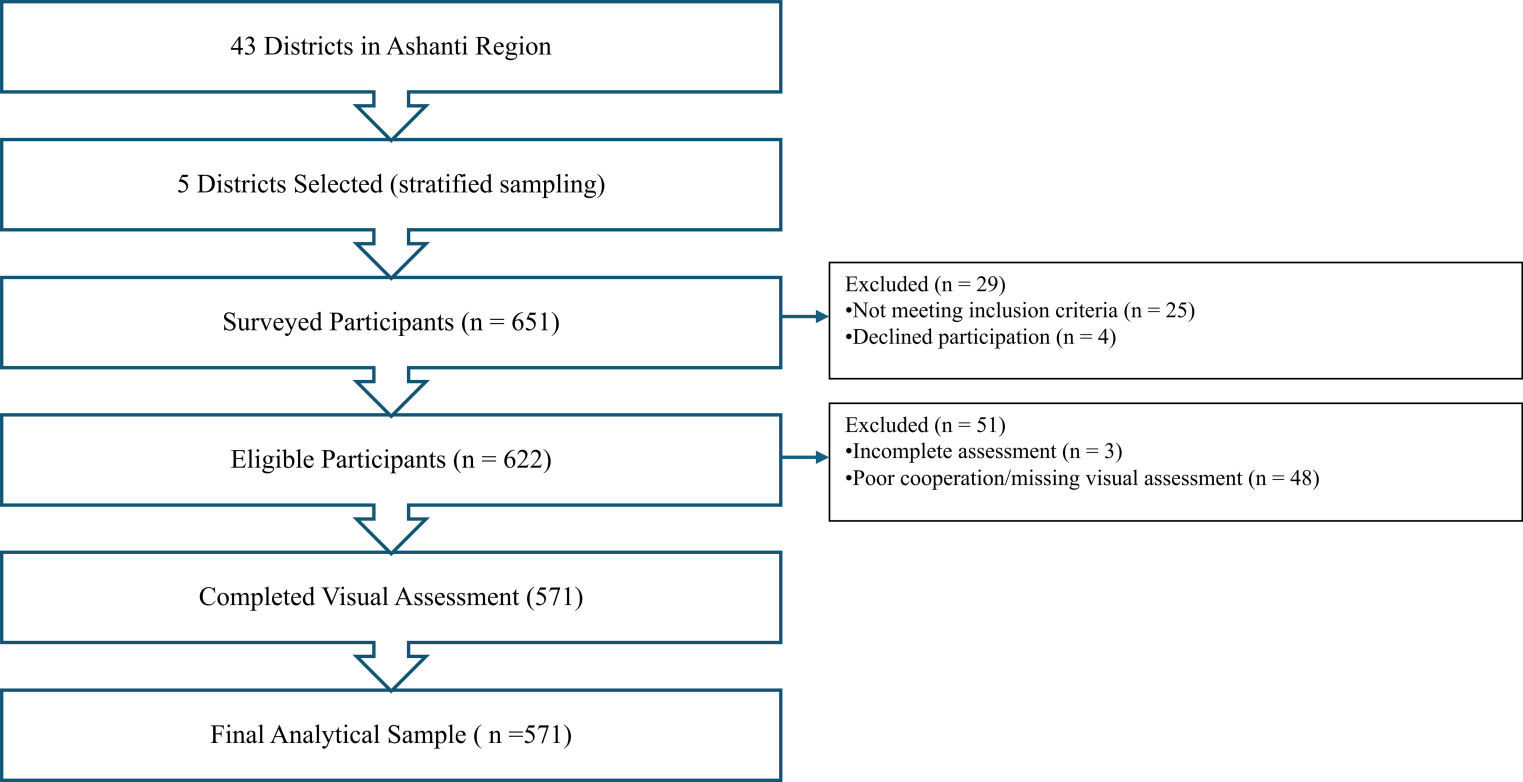
Flow diagram of participant recruitment, eligibility, visual assessment completion, and inclusion in descriptive and inferential analyses.

The study surveyed 651 participants; however, only 622 met the eligibility criteria, while some 51 participants (8.20% of the total sample) could not complete the vision assessments and were therefore excluded from analyses (see **Figure 1**).

### 2.6 Eligibility criteria

Eligibility required residence within the study area at the time of data collection. Written informed consent was obtained from participants aged 18 years and above, while parental or guardian consent was obtained for those below 18 years.

### 2.7 Study variables

The primary outcome was VI, assessed by a clinical team led by an optometrist during ophthalmic examination. Independent variables included child- and guardian-level characteristics. Child variables comprised age, sex, ethnicity, education level, settlement type, health insurance status, birth term, and ocular/systemic history. Guardian variables included marital status, employment status, income level, education level, smoking and alcohol use, and family history of ocular disease.

### 2.8 Data collection

#### 2.8.1 Community entry

Community entry was facilitated through collaboration with assembly members, district health directorates, heads of information centers, and community opinion leaders. Public announcements were made through community information centers, and mobilizers supported participant engagement and logistics, including the provision of venues and seating arrangements. Communities were informed about the study objectives, inclusion criteria, data collection sites, and scheduled dates and times. Data collection was conducted during weekends and public holidays to maximize participation. In addition, assembly members conducted household visits to encourage enrolment.

#### 2.8.2 Study questionnaire

A structured questionnaire was administered to guardians to obtain sociodemographic and household information. Information collected included child characteristics such as age, sex, birth term, and educational level, as well as guardian-related variables including age, educational level, ethnicity, marital status, employment status, monthly income, health insurance status, smoking, and alcohol use.

#### 2.8.3 Operational definition

Distance VI was defined as presenting visual acuity worse than 6/12 (Snellen) or >0.3 logMAR in the better-seeing eye. Near VI was defined as N6 or worse (0.8M or worse), based on ICD-11 criteria (23). Presenting distance VI was further classified as no VI (better or equal to 0.3), mild (worse than 0.3 but better or equal to 0.5 logMAR), moderate (worse than 0.5 but better or equal to 1.0 logMAR), severe (worse than 1.0 but better or equal to 1.3 logMAR), and blindness (worse than 1.3 logMAR).

#### 2.8.4 Visual acuity assessment

Visual acuity assessment was conducted using age-appropriate standardized tools under daylight conditions by trained final-year optometry students, who underwent a three-week pre-study training to ensure measurement consistency. Children aged <4 years or those with learning difficulties were assessed using LEA Grating^®^ paddles at 57 cm and 114 cm, first binocularly and then monocularly where possible. Children aged 4–7 years were assessed using LEA Symbols^®^ charts at 3 m, while those aged ≥8 years were tested using Sloan ETDRS charts at 3 m. Near vision was assessed at 40 cm using LEA Symbols^®^ near charts for children aged ≥4 years. Visual acuity results were recorded in LogMAR units for distance and M notation for near vision (24), with age-appropriate adjustments applied for children under 4 years (25). The better-seeing eye was used for classification of VI; when both eyes had equal acuity, the right eye was selected. For children under 4 years who could not complete monocular testing, binocular visual acuity was recorded. Thirteen children in this age group were unable to comply with monocular testing due to restlessness. No participant presented with pre-existing refractive correction. Near vision testing could not be completed in some very young children due to inability to identify optotypes.

#### 2.8.5 Ocular assessment

Ophthalmoscopy was performed on all participants to screen for ocular abnormalities. Fundus imaging was conducted using the Horus DEC200 fundus camera (MiiS, Hsinchu, Taiwan) to assess retinal pathology.

#### 2.8.6 Refractive assessment

Non-cycloplegic objective refraction was performed on participants using the PlusOptiX A12R autorefractor (PlusOptiX GmbH, Nuremberg, Germany), which is noted for its good sensitivity and specificity in identifying refractive errors without cycloplegia among children, especially in community settings (26–28). Subjective refraction was subsequently performed using trial lenses and pediatric frames to determine the need for correction.

### 2.9 Data analysis

Data analysis was performed using IBM SPSS Statistics version 27.0 (IBM Corp., Armonk, NY, USA). Descriptive, bivariate, and multivariable logistic regression analyses were conducted to determine the prevalence of visual impairment and its associated factors. Descriptive statistics were used to summarise the data, with categorical variables presented as frequencies and percentages, and continuous variables expressed as means and standard deviations (SD). Confidence intervals were calculated using the exact binomial (Clopper-Pearson) method. Participants with incomplete or poor visual assessment were excluded from analyses. Also, missing data were excluded from analyses. Inferential analysis was conducted using logistic regression to examine associations between VI and independent variables such as age, sex, ethnicity, settlement type, birth term, chronic systemic condition, and socioeconomic factors, identified in similar studies (5, 21, 29–34). Variables were entered into a bivariate model to explore their relationship to VI and adjusted in a multivariate model against potential confounders. Multicollinearity among independent variables was assessed using variance inflation factors (VIF), with all values below 2, indicating no evidence of multicollinearity. Model fitness was evaluated using the Hosmer–Lemeshow goodness-of-fit test (χ² = 8.03, df = 8, p = 0.43), which indicated adequate model fit. The predictive performance of the model was assessed using the receiver operating characteristic (ROC) curve, yielding an area under the curve (AUC) of 0.87, demonstrating good discriminatory ability in distinguishing participants with and without visual impairment. Statistical significance was set at p ≤ 0.05.

### 2.10 Ethical consideration

Ethical approval was obtained from the Committee on Human Research, Publication and Ethics (CHRPE), Kwame Nkrumah University of Science and Technology (CHRPE/AP/1245/24). Administrative permissions were secured from district assemblies prior to data collection. Written informed consent was obtained from parents or guardians, and from participants aged 18 years and above. Verbal assent was obtained from participants using age-appropriate explanations of study procedures at each stage of assessment. The study adhered to the principles of the Declaration of Helsinki, and participants were informed of their right to withdraw at any stage without consequence. Participants identified with any abnormality were further referred to the nearest eye health facility.

## 3 Results

### 3.1 Description of the sample

Most participants were aged 5–9 years (220, 38.5%) and 10–14 years (209, 36.6%), while 95 (16.6%) were aged 0–4 years and 47 (8.2%) were 15–20 years. Slightly more participants were female (306, 53.6%) than male (265, 46.4%). The majority resided in peri-urban areas (345, 60.4%), followed by urban (145, 25.4%) and rural settings (81, 14.2%). (See **Table 1**).

**Table 1:** Description of the sample.

Child variables	n	%
Total	571	100.0
Sex		
Male	265	46.4
Female	306	53.6
Age in years (mean ± SD)	8.88 ± 4.11	
Age in years (cat)		
0 to 4	95	16.6
5 to 9	220	38.5
10 to 14	209	36.6
15 to 20	47	8.2
Type of settlement		
Rural	81	14.2
Peri-urban	345	60.4
Urban	145	25.4

### 3.2 Prevalence and patterns of distribution of visual impairment

**Figure 2:**
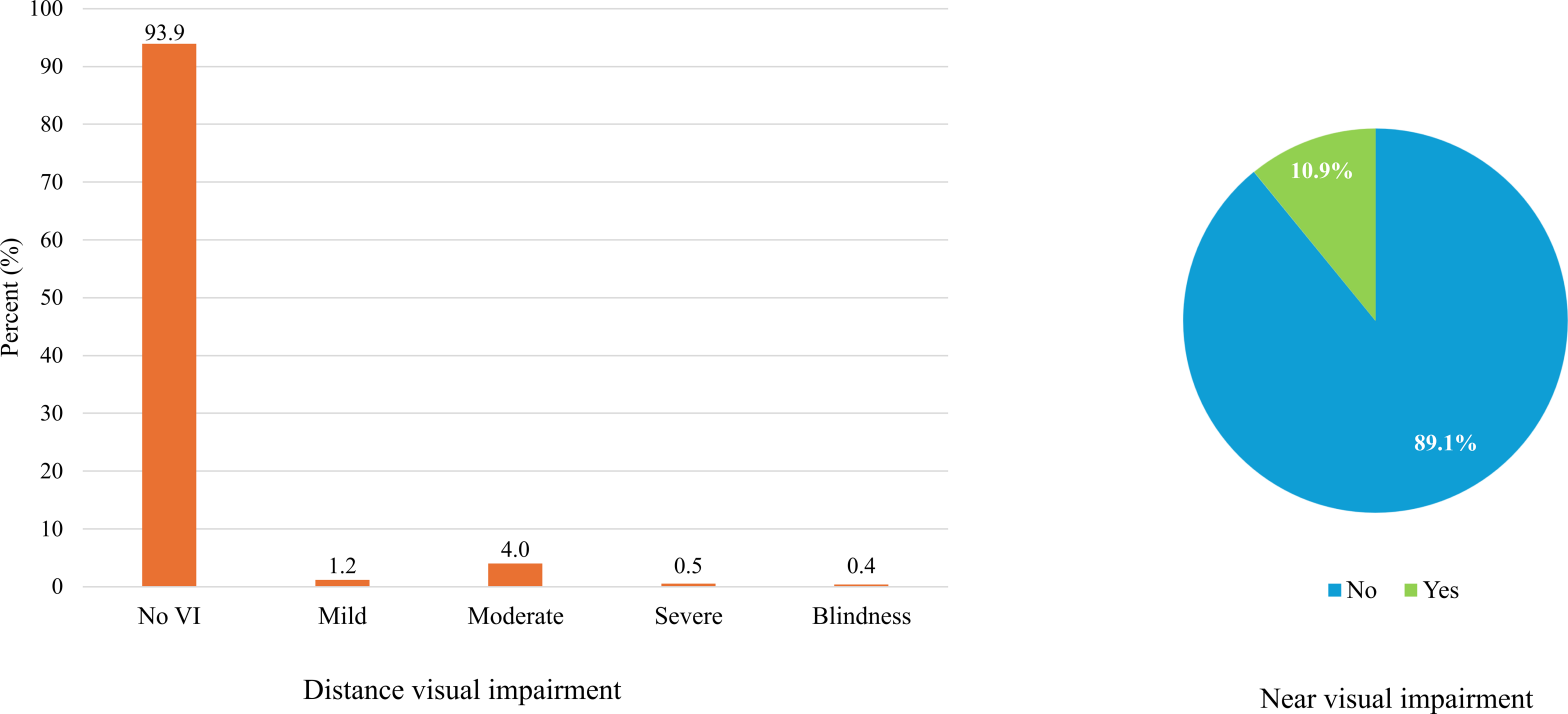
The proportion of distance VI (left) and near visual impairment (right).

Overall, the prevalence of VI in the study population was 35/571 (6.1%; 95% CI: 4.3-8.4). The prevalence of VI was marginally higher among females (20/306, 6.5%; 95% CI: 4.0-9.9) compared to males (15/265, 5.7%; 95% CI: 3.2-9.2). A marked age gradient was observed, with the highest prevalence in children aged 0–4 years (21/95, 22.1%; 95% CI: 14.2-31.8), declining substantially in older age groups. By settlement type, VI was more common in peri-urban areas (26/345, 7.5%; 95% CI: 5.0-10.8) compared to urban (7/145, 4.8%; 95% CI: 2.0-9.7) and rural settings (2/81, 2.5%; 95% CI: 0.3-8.6). Ethnic differences were evident, with higher prevalence among non-Akan groups (12/117, 10.3%; 95% CI: 5.4-17.2) compared to Akan children (23/450, 5.1%; 95% CI: 3.3-7.6). Clinically, VI was notably higher among children with a previous ocular condition (5/23, 21.7%; 95% CI: 7.5-43.7) and those born pre-term (2/14, 14.3%; 95% CI: 1.8-42.8), while differences by health insurance status were minimal. Socioeconomic factors showed modest variation, with higher prevalence among children of married guardians (29/410, 7.1%; 95% CI: 4.8-10.0) and those whose guardians were unemployed (8/89, 9.0%; 95% CI: 4.0-17.0), whereas guardian education level showed little difference (see **Table 2**). Of note, the proportionate distribution of the severity of participants with distance VI were 1.2% mild (n = 7), 4.0% moderate (n = 23), 0.5% severe (n = 3) and 0.4% blind (n = 2). With respect to near visual impairments dichotomized as yes and no, 89.1% (n = 451) had no near VI, whiles 10.9% had near VI (n = 55) (see **Figure 2**).

**Table 2:** Prevalence of visual impairment.

Variables	Overall	VI present		VI absent	
	n (%)	n (%)	95% CI	n (%)	95% CI
Child variables					
Total	571 (100.0)	35 (6.1)	4.3-8.4	536 (93.9)	91.6-95.7
Sex					
Male	265 (46.4)	15 (5.7)	3.2-9.2	250 (94.3)	90.8-96.8
Female	306 (53.6)	20 (6.5)	4.0-9.9	286 (93.5)	90.1-96.0
Age in years (mean ± SD)	8.88 ± 4.11	5.00 ± 4.99		9.13 ± 3.92	
Age in years (cat)					
0 to 4	95 (16.6)	21 (22.1)	14.2-31.8	74 (77.9)	68.2-85.8
5 to 9	220 (38.5)	8 (3.6)	1.6-7.0	212 (96.4)	93.0-98.4
10 to 14	209 (36.6)	4 (1.9)	0.5-4.8	205 (98.1)	95.2-99.5
15 to 20	47 (8.2)	2 (4.3)	0.5-14.5	45 (95.7)	85.5-99.5
Type of settlement					
Rural	81 (14.2)	2 (2.5)	0.3-8.6	79 (97.5)	91.4-99.7
Peri-urban	345 (60.4)	26 (7.5)	5.0-10.8	319 (92.5)	89.2-95.0
Urban	145 (25.4)	7 (4.8)	2.0-9.7	138 (95.2)	90.3-98.0
Ethnicity (n = 567) ^†^					
Akan	450 (79.4)	23 (5.1)	3.3-7.6	427 (94.9)	92.4-96.7
Others	117 (20.6)	12 (10.3)	5.4-17.2	105 (89.7)	82.8-94.6
Birth term (n = 457) ^†^					
Full-term	443 (96.9)	25 (5.6)	3.7-8.2	418 (94.4)	91.8-96.3
Pre-term	14 (3.1)	2 (14.3)	1.8-42.8	12 (85.7)	57.2-98.2
Health insurance (n = 568) ^†^					
No	57 (10.0)	4 (7.0)	1.9-17.0	53 (93.0)	83.0-98.1
Yes	511 (90.0)	31 (6.1)	4.2-8.5	480 (93.9)	91.5-95.8
Previous ocular condition					
No	548 (96.0)	30 (5.5)	3.7-7.7	518 (94.5)	92.3-96.3
Yes	23 (4.0)	5 (21.7)	7.5-43.7	18 (78.3)	56.3-92.5
Chronic systemic condition (n = 567) ^†^					
No	534 (94.2)	32 (6.0)	4.1-8.4	502 (94.0)	91.6-95.9
Yes	33 (5.8)	3 (9.1)	1.9-24.3	30 (90.9)	75.7-98.1
Family with ocular condition					
No	514 (82.6)	28 (5.4)	3.6-7.7	443 (86.2)	82.9-89.1
Yes	108 (17.4)	7 (6.5)	2.6-12.9	93 (86.1)	78.1-92.0
Guardian level of education (n = 567) ^†^					
No formal education	69 (12.2)	4 (5.8)	1.6-14.2	65 (94.2)	85.8-98.4
Formal education	498 (87.8)	31 (6.2)	4.3-8.7	467 (93.8)	91.3-95.7
Guardian marital status (n = 566) ^†^					
Not Married	156 (27.6)	5 (3.2)	1.0-7.3	151 (96.8)	92.7-99.0
Married	410 (72.4)	29 (7.1)	4.8-10.0	381 (92.9)	90.0-95.2
Guardian work status (n = 568) ^†^					
Unemployed	89 (15.7)	8 (9.0)	4.0-17.0	81 (91.0)	83.1-96.0
Employed	479 (84.3)	27 (5.6)	3.7-8.1	452 (94.4)	92.0-96.3
Guardian income (n = 482) ^†^					
<GHC3000	443 (91.9)	25 (5.6)	3.7-8.2	418 (94.4)	91.8-96.3
≥GHC3000	39 (8.1)	3 (7.7)	1.6-20.9	36 (92.3)	79.1-98.4
Guardian consume alcohol (n = 569) ^†^					
No	550 (96.7)	35 (6.4)	4.5-8.7	515 (93.6)	91.3-95.5
Yes	19 (3.3)	0 (0.0)	0-17.6	19 (100.0)	82.4-100.0
Guardian smokes (n = 569) ^†^					
No	569 (100.0)	35 (6.2)	4.3-8.5	534 (93.8)	91.5-95.7
Yes	0 (0.0)	0 (0.0)	0-100.0	0 (0.0)	0-100.0

^†^ n ≠ 571 as some responses were missing; Others: Guan, Wala, Ga/dangbe, Gonja, Mossi, Banda, Busanga, Konkomba, Sisala, Gurma, Nafana, Dagati, Dagomba, Grusi.

### 3.3 Causes of visual impairment

**Figure 3:**
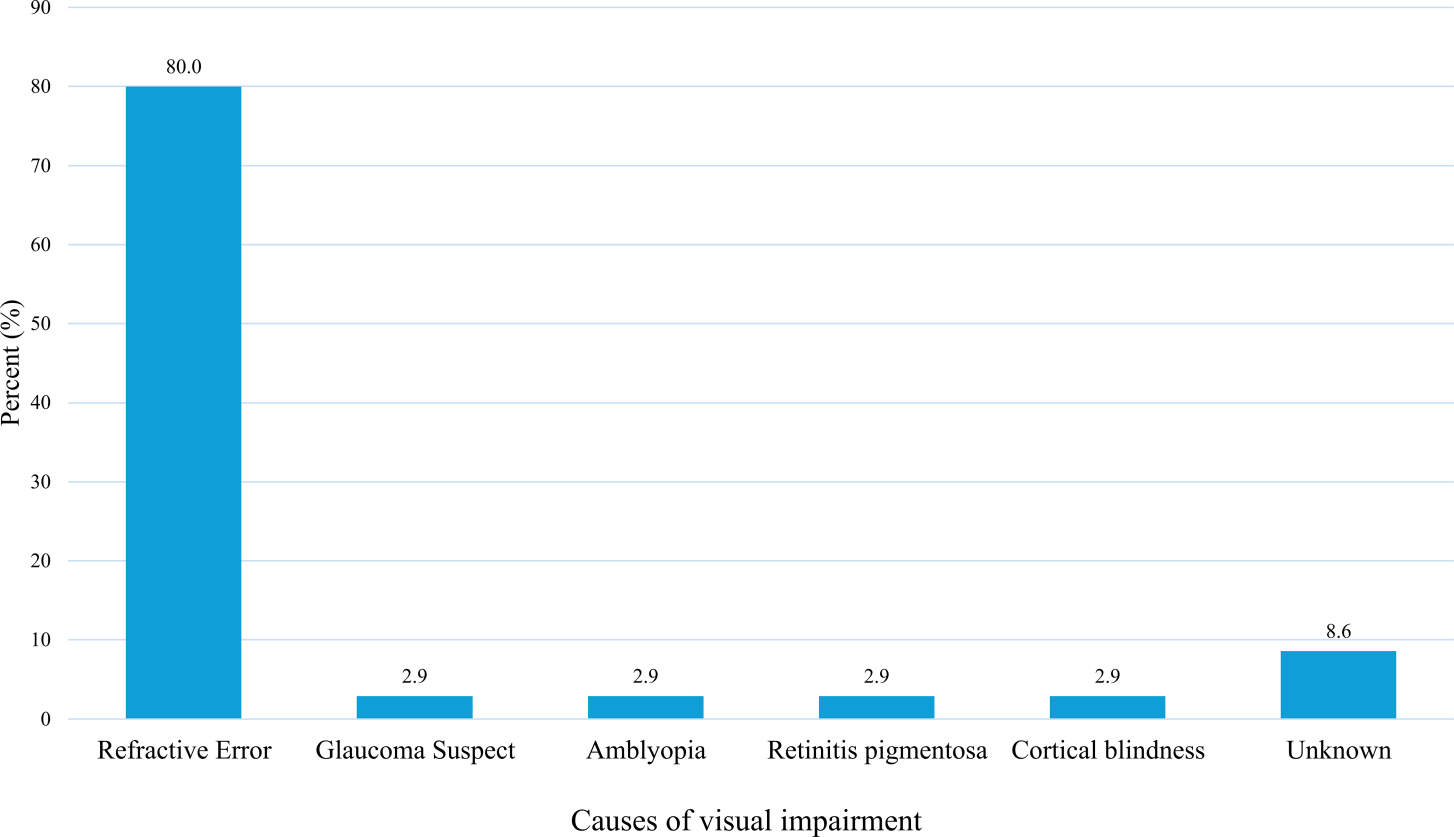
Proportionate distribution of the causes of visual impairments in the sample.

Refractive error was the predominant cause of VI, accounting for 28 (80.0%) cases, indicating that the majority of VI in this population is potentially correctable. The remaining causes were each relatively rare: glaucoma suspect 1 (2.9%), amblyopia 1 (2.9%), retinitis pigmentosa 1 (2.9%), and cortical blindness 1 (2.9%). In 3 (8.6%) cases, the cause was unknown (see **Figure 3**).

### 3.4 Visual impairment and associated factors

**Table 3** shows bivariate and multivariate logistic regression analysis of factors associated with VI among the study sample. In the bivariate (univariate) logistic regression analysis, increasing age was associated with lower odds of VI (COR = 0.73, 95% CI: 0.66–0.82, p < 0.001). Children from other ethnic groups had lower odds compared to Akan children (COR = 0.47, 95% CI: 0.23–0.98, p = 0.043). Children with a previous ocular condition had lower odds compared to those without (COR = 0.21, 95% CI: 0.07–0.60, p = 0.004). Other variables, including sex, type of settlement, guardian education, birth term, health insurance status, chronic systemic condition, family ocular history, guardian marital status, work status, and income level, were not significantly associated with VI at the bivariate level (p > 0.05). After further adjusting for all other variables in the multivariate logistic regression analysis, only increasing age (AOR = 0.68, 95% CI: 0.57–0.82, p < 0.001) and children from other ethnic groups as compared to those of Akan ethnicity (AOR = 0.10, 95% CI: 0.03–0.37, p <0.001) remained significantly associated with lower odds of VI (see **Table 3**).

**Table 3:** Bivariate and multivariate regression of factors associated with VI.

Variables	Univariate logistic regression			Multiple logistic regression		
	COR	95% CI	p-value	AOR	95% CI	p-value
Sex						
Male	ref					
Female	0.86	0.43-1.71	0.664	1.07	0.36-3.20	0.909
Age	0.73	0.66-0.82	<0.001*	0.68	0.57-0.82	<0.001*
Type of settlement						
Rural	ref					
Peri-urban	0.50	0.10-2.46	0.393	4.84	0.29-79.89	0.270
Urban	1.61	0.68-3.79	0.279	7.21	0.65-79.45	0.107
Guardian’s level of education						
No formal education	ref					
Formal education	0.93	0.32-2.72	0.894	0.30	0.05-1.83	0.190
Ethnicity						
Akan	ref					
Others	0.47	0.23-0.98	0.043*	0.10	0.03-0.37	<0.001*
Birth term						
Full-term	ref					
Pre-term	0.36	0.08-1.69	0.195	0.21	0.03-1.44	0.113
Participant health insurance						
No	ref					
Yes	1.17	0.40-3.44	0.777	2.00	0.18-6.91	0.920
Previous ocular condition						
No	ref					
Yes	0.21	0.07-0.60	0.004*	0.26	0.03-2.11	0.206
Chronic systemic condition						
No	ref					
Yes	0.64	0.19-2.20	0.476	0.18	0.02-2.21	0.180
Family previous ocular condition						
No	ref					
Yes	0.87	0.37-2.05	0.745	0.51	0.13-2.04	0.338
Guardian marital status						
Not Married	ref					
Married	0.44	0.17-1.15	0.092	0.12	0.01-1.14	0.064
Guardian work status						
Unemployed	ref					
Employed	1.65	0.73-3.77	0.231	0.00	0.00	1.000
Guardian income level						
** <**GHC3000	ref					
≥GHC3000	0.72	0.21-2.49	0.602	0.31	0.07-1.44	0.136

COR, crude odds ratio; AOR, adjusted odds ratio; CI, confidence interval; *represents statistical significance.

## 4 Discussion

This community-based cross-sectional study presents the burden and determinants of VI among the pediatric population in the Ashanti Region of Ghana. Nearly one in sixteen children was found to be visually impaired, with most cases classified as moderate to severe, and refractive errors identified as the leading cause. After statistical adjustment, increasing age and ethnicity were significantly associated with lower odds of VI.

VI in childhood has profound consequences, affecting educational attainment, cognitive development, social participation, and overall quality of life (35, 36). Despite these long-term impacts, evidence on the burden and determinants of childhood VI remains limited in many LMICs, where access to routine eye screening and pediatric eye care is often constrained (37, 38). In Ghana, particularly within the Ashanti Region, population-based data are scarce, limiting effective planning and intervention strategies. This study, therefore, aimed to address this gap by examining the prevalence and associated factors of VI among children. Children under 21 years were therefore recruited in accordance with the WHO’s definition of adolescents, as ranging from 10 to 19 years (39) and the recommendation to extend the upper age limits of pediatric definitions with Western Sub-Saharan populations favoring an upper limit of up to 19 years, while high-income North America largely favored the upper limit of 20 years (40). This suggests the differing age cutoffs for children globally. Moreover, similar population-based studies among children in Ghana have favored the upper age limit of 21 years (18, 21). This study hypothesized that a combination of demographic, clinical, and contextual factors influences VI in children.

In this study, approximately one in sixteen children (6.1%) had VI. This burden is higher than the global estimate of 3.82% (visual acuity ≤ 20/60) and the African estimate of 3.57% (visual acuity ≤ 20/40) reported in previous reviews (41). Conversely, estimates of childhood VI vary substantially across African settings, with reported prevalence ranging from 1.2% in South Africa (42) to 2.4% in Kenya (43), 7% in Nigeria (44) and Ethiopia (45), and up to 10.8% in Mozambique (46). Similarly, our estimates are relatively higher than previous studies conducted in Ghana, which have reported lower prevalence estimates, including 0.61% among children aged 0–9 years and 0.24% among adolescents aged 10–19 years (47), 3.9% among children aged 12–15 years (21), and 3.5% among those aged 9–22 years (5). This pattern of variation in this study likely reflects differences in study design, sampling frames, age distributions, and case definitions, as well as the inclusion of both distance and near VI in the present study. Also, the high prevalence of near VI can be attributed to the accommodative anomalies that may be prevalent among the sample. Again, the higher prevalence of near VI relative to distance VI can be attributed to the observation that younger children are mostly hyperopic and gradually become emmetropic as they get older (48, 49). Further studies to explore the accommodative function of children in the present population are recommended.

In the current study, refractive error was identified as the leading cause of VI, accounting for more than half (80.0%) of VI cases. Even though it remains the most common and readily treatable cause of childhood VI, other studies in Ghana have similarly reported high prevalence of refractive error 25.6% (33), 78.7% (21) and although other studies have shown lower prevalences of 3.7% (5) and 1.8% (50), refractive error was the primary cause of VI. This finding highlights uncorrected refractive errors as a persistent public health hurdle, especially in LMICs (7, 38). This finding was similar in school settings (5, 30, 51) as well as community studies (43, 52). However, of note, school studies generally reported on some participants wearing spectacle corrections when measuring presenting VI, but participants in this study did not present with corrective wear nor report having them. This may reflect the demand on the visual system in school settings, allowing for early detection and management. There is a need to bridge critical gaps, such as access to routine vision screening, especially in communities and schools, and affordable refractive services to reduce the burden of visual impairment.

Age was significantly associated with VI. With increasing age, there was a decreased likelihood of VI. There are scarce comparative studies in Ghana on VI among children, especially involving those aged younger than five years. Abu et al. (21), however, reported no association between VI and age among pupils aged 9 to 22 years. Awan et al. (53) observed a positive association between VI and age among 5 to 20-year-old school children, contrary to this study’s finding. The observed pattern in this study may be due to biological and developmental reasons, as the visual system undergoes critical periods of maturation during infancy and early childhood (54). Another factor is compliance issues, where younger children may have had difficulties comprehending test instructions, sustaining attention, or cooperating fully during visual acuity assessments, which may have led to apparent reductions in measured visual performance, resulting in overestimation of VI. Nonetheless, this finding highlights the importance of timely identification and management of ocular conditions, which are essential to prevent long- term deficits in cognitive, motor, and educational outcomes.

The finding that ethnicity was significantly associated with VI was similar to other studies that compared different ethnicities and races (55, 56), citing that the association was most likely due to access and uptake of eye health services, though another study found no association between ethnicity and VI (34). The current study found that those of other ethnicities had lower odds of presenting with VI than Akans, although VI was more prevalent amongst non-Akan children, suggesting confounding by factors such as age. Ultimately, children of other ethnicities having lower odds of VI could be attributed to Akans making up the majority of the study sample and therefore bearing the absolute burden of VI.

This study also found that a prior diagnosis of ocular disease was initially associated with lower odds of VI. This likely reflects differential access to eye care, with children previously diagnosed with ocular conditions more likely to have had contact with eye care services, enabling earlier detection, treatment, and follow-up (21). Nonetheless, this was not statistically significant after adjusting for other variables. Collectively, these results highlight the imperative for early vision screening among children and addressing the burden of preventable VI, which accounts for the majority of VI cases.

### 4.1 Study strengths and limitations

This study has some limitations. First, voluntary participation introduces the potential for selection bias, as caregivers with concerns about their children’s vision may have been more likely to participate, potentially inflating prevalence estimates. As such, results should not be interpreted as representative of the broader population of children in similar settings. Second, cycloplegic refraction, though the gold standard for identifying refractive errors in children, was not used due to the large sample and nature of the study sites, and this could influence refractive results, though the alternative use of the Plusoptix A12 R without cycloplegia has been found to be equally robust. Finally, the cross-sectional design precludes causal inference. This study’s strengths lie in the stratified sampling of districts to increase representativeness, a community-based design that reveals the true burden of VI by allowing participation of all, including marginalized groups, and the involvement of children under 5, for whom there is limited data on VI.

## 5 Conclusion

In summary, this study highlights the significant burden of VI, especially among children, emphasizing the urgent need for intervention. Future research would benefit from a longitudinal approach focusing on strategies to guide and inform national policies.

## Data Availability

The dataset supporting the conclusion of this paper is available from the corresponding author upon reasonable request.
